# Prevalence of drug abuse and it’s perceived effect on the mental health and academic performance of secondary school students in Bauchi State Nigeria.

**DOI:** 10.1192/j.eurpsy.2025.579

**Published:** 2025-08-26

**Authors:** R. M. Bawa, H. I. Gomma, A. A. Abdullateef

**Affiliations:** 1 Psychiatric Nursing, Federal university of health sciences, Azare, Bauchi; 2 Nursing sciences, Ahmadu Bello University Zaria, Kaduna; 3 Psychiatry, college of medical sciences, Ahmadu Bello University, zaria, Nigeria

## Abstract

**Introduction:**

The study was designed to determine the prevalence of drug abuse and its perceived effect on the mnetal health and academic performance of secondary school students in Bauchi State, Nigeria.

**Objectives:**

**Objectives of the Study**

The aim of this study is to examine the prevalence of drug abuse and its perceived effects on the mental health and academic performance of secondary schools student in Bauchi state. The specific objectives are:

1. To determine the prevalence of drug abuse among secondary school students in Bauchi metropolis.
2. To identify the perceived drugs commonly abused by students in secondary schools.
3. To investigate perceived reasons secondary school students abuse drug in Bauchi metropolis.
4. To investigate the perceived negative effect of drug abuse on the mental health and school performance of the students in secondary schools of Bauchi metropolis.

**Methods:**

The study adopted a cross sectional descriptive design. Multistage sampling procedure was used to select 26 Secondary Schools in Bauchi state. The schools have a combined population of 11,439 students. The instrument for Data collection was a WHO Youth Drug Survey (WHOYDSQ) and drug abuse screening test (DAST) adapted questionnaire and a sample size of 399 was obtained using Yamane formula. The reliability of the instrument was established using a test and re-test. Data generated analysed using frequency distribution tables, cross tabulation and chi square.

**Results:**

Out of 399 copies of the questionnaire distributed, 372 were correctly filled and analyzed. Majority (80%) of the respondents were between 18-20 years. More than half, 208 respondents (55.9%) reported to have used drug for non-medical reasons once or more in the past one year. The commonest substances abused were codeine, cough syrup, cannabis and tramadol. More than half of the users of each of the substances take it occasionally. Among the respondents, 42.8% who used psychoactive substances were introduced to the substance by their friends. Major reasons for using psychoactive substance include reduction of stress (37.1%), out of curiosity (28%) and memory improvement and retention (26.6%). There was significant difference (p < 0.05) in the academic performance of the respondents that abuse drug and those that do not.

**Conclusions:**

In conclusion the prevalence of substance abuse among Secondary School students was high (55.9%) as such government, parents, teachers, stakeholders and the community leaders needs to join hands to fight and address the problem.

**Disclosure of Interest:**

None Declared

